# Identification of eIF4ET binding partners in *C. elegans*

**DOI:** 10.17912/micropub.biology.002209

**Published:** 2026-08-17

**Authors:** Judith Tafur, Angelica Morales Cueva, Nicole E. Familiari, Weronika E. Stachera, Jeffrey B. Woodruff

**Affiliations:** 1 Cell Biology, The University of Texas Southwestern Medical Center, Dallas, TX, United States

## Abstract

The eIF4ET family protein IFET-1 is needed for oocyte development and long-term storage in *C. elegans* . How IFET-1 plays multiple roles in oocyte biology is unclear. Using pull-downs and mass spectrometry, we identified proteins that interact with IFET-1 . We show that three IFET-1-interacting partners— PENR-1 , SQD-1 , and LSM-4 —are essential for producing viable oocytes in *C. elegans* females.

**Figure 1. IP-mass spectrometry identifies diverse interacting partners of IFET-1 in C. elegans females:** A. Top, confocal image of a day 2 adult feminized worm gonad expressing IFET1::mSCarlet::AID*::3xFlag. Bottom, schematic of IFET-1 ::3XFlag pulldown assay and mass spectrometry analysis of interacting proteins. B. Volcano plot of protein abundances in pulldown experiment using ifet-1 ::mScarlet::AID*::3xFLAG; fem-1 ( hc17 ) adult females vs *fem-1 ( hc17 )* worms as a control. Y-axis represents the –Log transformation of the obtained p-values. X-axis represents fold enrichment in abundance (FLAG-tag vs. control; n = 3 replicates). See Extended Data Set 1 for more information. C. Log _2_ transformed fold changes of selected top IFET-1 pulldown hits grouped by gene ontology, molecular function, and biological process categories. Note: LSM-4 was identified in the IFET-1 ::3xFLAG pull-down and not in the control; thus, a fold-change score could not be determined. D. Feminized worms were treated with RNAi, then mated with untreated males. Shown is embryo viability post mating (mean ±95% C.I; >25 offspring counted from n=25 mothers (control), n=16 ( *penr-1* ), n=9 ( *sqd-1* ), n=7 ( *lsm-4* ), n=9 ( *cid-1* ), n=5 ( *patr-1* ), n=4 ( *edc-3* ), n=6 ( *fndc-1* ), n=7 ( *sup-26* ), n=10 ( *CELE_W01F3.2* ), n=5 ( *F26E4.3* ), n=5 ( *CELE_F56G4.6* ), n=5 ( *CELE_T13H5.8* ), and n=5 ( *acly-1* )). p-value=0.0007 (control vs. *penr-1* ), p-value<0.0001 (control vs. *sqd-1 )* and p-value<0.0001 (control vs. *lsm-4* ) *.* P values from one-way ANOVA followed with Dunnet's multiple comparisons test.

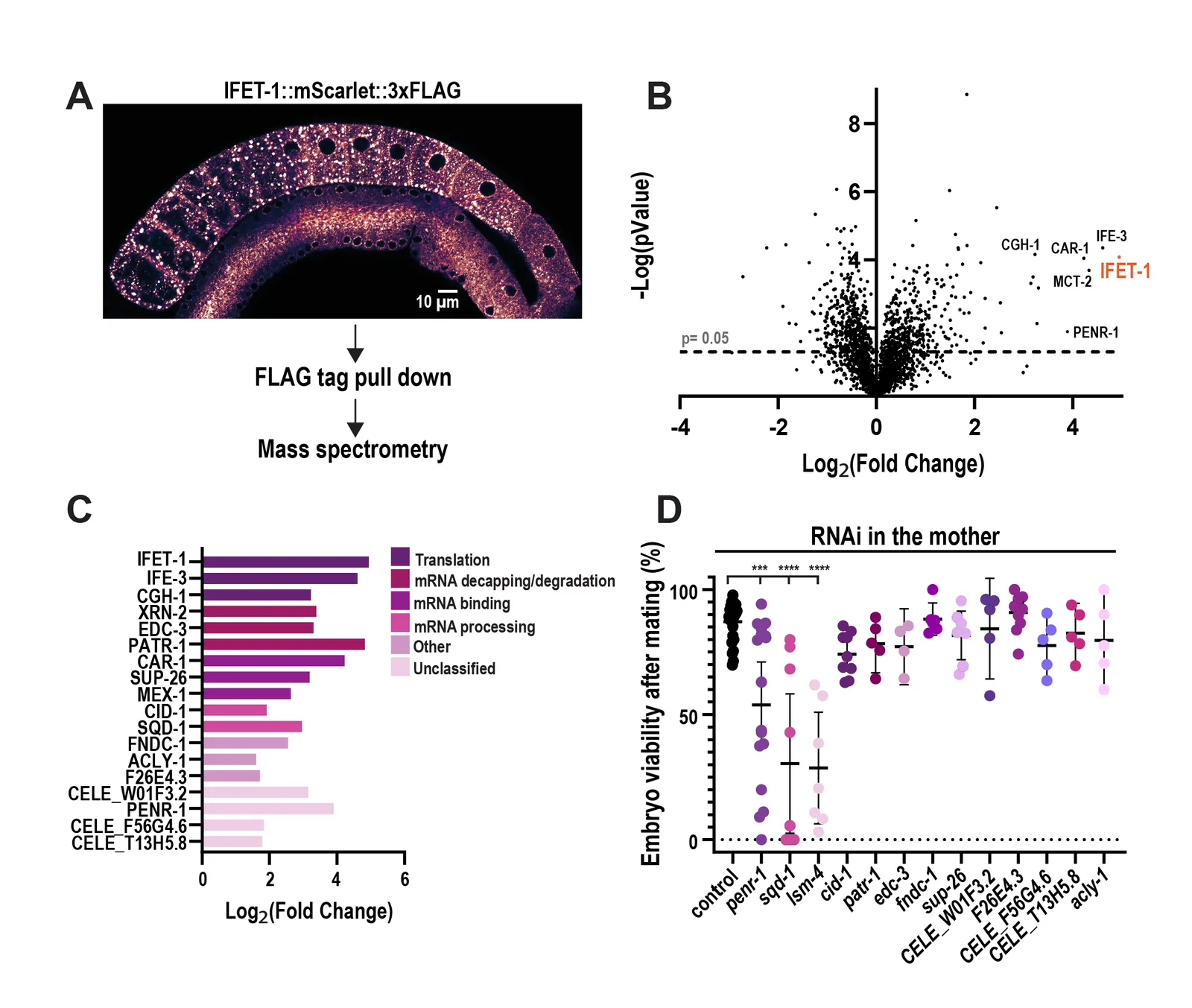



## Description

In *C. elegans* , RNA-rich biomolecular condensates called germ granules are required for germ cell proliferation and sperm/oocyte development (Huggins et al., 2020; Kawasaki et al., 2004; Spike et al., 2008a; Spike et al., 2008b). Germ granules are multi-phase compartments, which allows for localized concentration of distinct protein pools and specialized functions in RNA processing and export from the nucleus. One important sub-compartment is the P-body, which contains the dead box helicase DDX6 ( CGH-1 in *C. elegans* ), the LSM domain-containing protein LSM14 ( CAR-1 ), the translation initiation factor eIF4E ( IFE-3 ), and the eIF4E Transporter ( IFET-1 )(Huang et al., 2025). IFET-1 enables selective repression of certain developmental transcripts, while promoting translation of others encoding cytoskeletal proteins (Bhatia et al., 2025; Huggins et al., 2020; Sengupta et al., 2013). It is likely that IFET-1 acts together with other P-body proteins to execute these diverse functions. For example, eIF4E Transporter interacts directly with DDX6 and LSM14 (Ozgur et al., 2015; Brandmann et al., 2018), and IFET-1 has been pulled down with IFE-3 (Li et al., 2009). Thus, we reasoned that by identifying interacting partners of IFET-1 , we could discover new factors that are essential for translational control during oocyte development and storage.

To identify IFET-1 binding partners, we used FLAG-Trap beads (anti-DYKDDDK agarose) to pull down FLAG-tagged IFET-1 ( IFET-1 ::mScarlet::3XFLAG) from adult female worms grown in liquid culture. In this line, IFET-1 localized to cytoplasmic puncta in oocytes and around the nuclei in the syncytial gonad, as expected ( Fig. 1A ). This construct was verified to be functional in our prior study (Bhatia et al., 2025). Female worms expressing untagged IFET-1 were used as a control. We analyzed co-purifying proteins by mass spectrometry and defined positive hits as being significantly enriched in the positive sample (enrichment >3-fold, p value <0.05; three replicates).

Top hits included IFET-1 and known IFET-1 interactors such as IFE-3 , CAR-1 , and CGH-1 (Huggins et al., 2020; Sengupta et al., 2013), validating our approach ( Fig. 1B ). GO term analysis revealed that IFET-1 's top binding partners consisted mostly of proteins involved in translation or mRNA regulation, consistent with reported functions for IFET-1 ( Fig. 1C ) (Bhatia et al., 2025; Huggins et al., 2020; Sengupta et al., 2013). To find novel proteins that promote oocyte production and viability, we used RNAi to knock down 13 of the top hits not known previously to be IFET-1 interactors ( Fig. 1D ). Of these, only knockdown of *lsm-4* , *sqd-1 ,* and *penr-1* reduced the percentage of viable embryos laid by RNAi-treated female worms mated with untreated males ( Fig. 1D ). LSM-4 and SQD-1 are both RNA binding proteins that have been characterized in oocyte development (Cornes et al., 2015; Erdmann et al., 2024). PENR-1 was originally identified in a high-throughput screen for genes that affect expression of the transcription factor PHA-4 during *C. elegans* embryogenesis (Green et al., 2024). The exact role of PENR-1 protein in germline formation remains unknown.

In summary, we identified interacting partners of IFET-1 , which could potentially explain how IFET-1 plays multiple roles in germline development and translational control. IFET-1 likely suppresses translation of specific transcripts by binding to IFE-3 —the cap-binding eIF4E initiation factor—and blocking its ability form a complex with eIF4G, thus preventing ribosome recruitment (Peter et al., 2015; Huggins et al, 2020). Some interactors, such as CAR-1 and CGH-1 , likely cooperate with IFET-1 in the P-body to sequester developmentally regulated mRNAs. Most of the other interactors have similar characterized roles in mRNA binding and processing. Interestingly, we identified unexpected interactors that do not have well-defined roles, one of which ( PENR-1 ) is also required for oocyte development. Future studies are needed to clarify how these IFET-1 partners could contribute to germline expansion and promotion of translation during oocyte storage.

## Methods

Worm husbandry

Worms were grown on Nematode Growth Media (NGM) plates and fed with standard OP50 *E. coli* . All worms were kept at 16°C for maintenance and 20°C for experiments. *fem-1* lines were shifted to 25°C for 2.5 days starting at L1 stage to feminize until adulthood at which point they were moved to 20°C. Experiments involving synchronization were done using 5% sodium hypochlorite and 5 M NaOH to remove all larvae and adult worms. Embryos were then rotated in M9 overnight to hatch.

IFET-1 pull down and mass spectrometry

*fem-1 ( hc17 )* and *fem-1 ; ifet-1* ::3XFLAG worms were grown at 16°C on large NGM plates for about a week until they became densely populated. The worms were then washed off with M9 PEG and added to large flasks containing S-Medium Complete as described in Wormbook Maintenance of *C. elegans* : Chapter 5. The liquid culture was grown in a 16°C shaker for about 4 days. The worms were then washed and synchronized using the same reagents described above. After rotating overnight, new liquid culture flasks were started using the synchronized L1 worms and grown in a 25°C shaker for 2.5 days. They were then transferred to 20°C to grow for a day.

The feminized adult worm samples were collected, pelleted, and flash frozen in PBS containing protease inhibitors. On the day of the pulldown experiment, ChromoTek DYKDDDDK Fab-Trap™ Agarose beads were equilibrated in low salt buffer (165mM KCl, 25 mM HEPES, 1% glycerol and 0.05% Tween 20). They were then blocked for 1 hour at 4°C with 3% milk in low salt buffer and washed 3 times with lysis buffer. While the beads blocked, the feminized worm pellets were thawed at 4°C in lysis buffer (50mM HEPES, 1mM EGTA, 100mM KCL, 1mM MgCl _2_ , 0.05% NP-40 and 1X protease inhibitors added fresh). They were then lysed by dounce homogenization and sonication at 35% amplitude. This lysate was then centrifuged at 17000 g for 25 minutes at 4°C. The lysate supernatant was then incubated on a rotator with the prepared beads for 10 minutes at room temperature and then at 4°C for 1 hour. The beads were sedimented and washed 3 times with low salt buffer. Samples were eluted with SDS and boiled for 5 min. They were then run on an SDS-PAGE gel for a few minutes so they would enter the gel, stained with Instant blue dye and carefully cut out to send to the mass spectrometry core facility.

For mass spectrometry, gel samples were digested overnight with trypsin (Pierce) after reduction and alkylation with DTT and iodoacetamide (Sigma-Aldrich). After solid-phase extraction cleanup with an Oasis HLB μElution plate (Waters), the resulting peptides were reconstituted in 2% (vol/vol) acetonitrile (ACN) and 0.1% trifluoroacetic acid in water. 1 μg of each sample was injected onto an Orbitrap Fusion Lumos mass spectrometer coupled to an UltiMate 3000 RSLCnano liquid chromatography system (Thermo Fisher Scientific). Samples were injected onto a 75 μm i.d., 75-cm-long EasySpray column (Thermo Fisher Scientific), and eluted with a gradient from 0 to 28% buffer B over 90 min. Buffer A contained 2% (vol/vol) ACN and 0.1% formic acid in water, and buffer B contained 80% (vol/vol) ACN, 10% (vol/vol) trifluoroethanol, and 0.1% formic acid in water. The mass spectrometer operated in positive ion mode with a source voltage of 2.5 kV and an ion transfer tube temperature of 300°C. MS scans were acquired at 120,000 resolution in the Orbitrap, and up to 10 MS/MS spectra were obtained in the Orbitrap for each full spectrum acquired using higher energy collisional dissociation (HCD) for ions with charges 2–7. Dynamic exclusion was set for 25 s after an ion was selected for fragmentation.

Raw MS data files were analyzed using Proteome Discoverer v3.0 (Thermo Fisher Scientific), with peptide identification performed using Sequest HT searching against the *C. elegans* reviewed protein database from UniProt along with the sequence of fluorescent mMaple protein. Fragment and precursor tolerances of 10 ppm and 0.6 D were specified, and three missed cleavages were allowed. Carbamidomethylation of Cys was set as a fixed modification, and oxidation of Met was set as a variable modification. The false discovery rate cutoff was 1% for all peptides.

RNAi Screen and viability assays

RNAi clones were obtained from the Ahringer library or cloned using the pL4440 empty vector plasmid and target DNA sequences of 300 bp ordered from TWIST biosciences. RNAi plates were made by growing the feeding clone containing bacteria on NGM plates containing 1mM IPTG and 100 μg/ml ampicillin. *fem-1 ( hc17 )* worms were synchronized at L1 stage, then grown on RNAi plates for 2.5 days at 25°C. Female adult worms were transferred to mating plates at a ratio of 1 female per 3 males, then incubated for 16 hours at 16°C. The number of embryos laid per mom was tallied and 2 days later the fraction of hatchlings was counted to obtain the viability of the laid eggs.

Microscopy

The microscope image was taken using a 40X silicone (NA 1.25) objective on a Nikon AX-R confocal microscope.

## Reagents

**Table 1: Worm Strains**

**Table:** 

Strain name	Genotype	Source
BA17	*fem-1 ( hc17 ) IV *	CGC
N2		CGC
JWD10	wrdSi3 [sun-1p::TIR1::F2A::mTagBFP2::AID*::NLS:: tbb-2 3'UTR] (II:0.77)	CGC
JWW255	fem-1 ( hc17 ) IV; ifet-1 ( dfw16 [ ifet-1 ::mScarlet_I::AID*::3xFlag]) III; wrdSi3 [sun-1p::TIR1::F2A::mTagBFP2::AID*::NLS:: tbb-2 3'UTR] (II:0.77)	Our lab

**Table 2: Plasmids and RNAi Feeding Clones**

**Table:** 

Plasmid Name	Parent Plasmid	Targeting Sequence
JWB 124	pL4440	Empty Vector
JWB 226	pL4440	*edc-3*
JWB 227	pL4440	*penr-1*
JWB 238	pL4440	*acly-1*
JWB 239	pL4440	*cid-1*
JWB 240	pL4440	*fndc-1*
JWB 241	pL4440	*sup-26*
JWB 242	pL4440	*sqd-1*
JWB 243	pL4440	*CELE_W01F3.2*
JWB 244	pL4440	*CELE_F56G4.6*
JWB 245	pL4440	*CELE_T13H5.8*
JWB 246	pL4440	*F26E4.3*
JWB 254	pL4440	*lsm-4*
JWB 264	pL4440	*patr-1*

## Data Availability

Description: Proteomic data and a list of meaningful IFET-1 interactors.. Resource Type: Dataset. DOI: https://doi.org/10.22002/je5p4-zyb48
